# Genome-wide identification and expression analysis of *calmodulin-like* (*CML*) genes in Chinese cabbage (*Brassica rapa* L. ssp*. pekinensis*)

**DOI:** 10.1186/s12864-017-4240-2

**Published:** 2017-11-02

**Authors:** Shanshan Nie, Minjuan Zhang, Lugang Zhang

**Affiliations:** College of Horticulture, Northwest A&F University, Yangling, 712100 Shaanxi People’s Republic of China

**Keywords:** *CML* gene, Chinese cabbage, Expression profiling, Stress response, Interaction network

## Abstract

**Background:**

Calmodulin-like (CML) proteins are a primary family of plant-specific Ca^2+^ sensors that specifically bind to Ca^2+^ and deliver a Ca^2+^ signal. CML proteins have been identified and characterized in many plant species, such as the model plant *Arabidopsis* and rice. Based on considerable evidence, the roles of CML proteins are crucial in plant growth and development and in the response to various external stimuli. Nevertheless, the characterization and expression profiling of *CML* genes in Chinese cabbage (*Brassica rapa* L. ssp*. pekinensis*) remain limited.

**Results:**

In this study, a genome-wide search and comprehensive analysis were performed, and a total of 79 *BrCML* genes were identified in Chinese cabbage. Gene structure analysis revealed that these *BrCML* genes contained two to four conserved EF-hand motifs. Phylogenetic analysis showed that CML homologs between Chinese cabbage and *Arabidopsis* shared close relationships. The identified *BrCML* genes were located across ten chromosomes and three different subgenomes of Chinese cabbage. Moreover, 126 pairs of orthologous *CML* genes were found among Chinese cabbage, *Arabidopsis* and *Brassica oleracea*. Expression analysis revealed that the expression of some *BrCML* genes was tissue-specific and that of some was susceptible to temperature stress. A putative interaction network of BrCML proteins was proposed, which suggested that BrCML2, BrCML6, BrCML15 and BrCML25 were co-expressed and might play roles in flower development and other relevant biological processes of Chinese cabbage.

**Conclusions:**

The results of this study increased the understanding and characterization of *BrCML* genes in Chinese cabbage, and will be a rich resource for further studies to investigate BrCML protein function in various developmental processes of Chinese cabbage.

**Electronic supplementary material:**

The online version of this article (10.1186/s12864-017-4240-2) contains supplementary material, which is available to authorized users.

## Background

Calcium (Ca^2+^), an essential secondary messenger in eukaryotic cells, plays major roles in many aspects of plant growth and development [1–3]. A variety of internal stimuli and external abiotic and biotic stresses, including temperature, light, drought, salinity, plant hormones and disease [1, 4], can induce variation in the level of cytoplasmic free Ca^2+^ and affect the movements of Ca^2+^ in plant cells. The stress signalling is sensed by unique Ca^2+^ sensors or Ca^2+^-binding proteins [3, 5, 6]. Ca^2+^-binding proteins binding to Ca^2+^ trigger their conformational changes, and the modulation of activity subsequently regulates downstream targets, thereby transmitting the Ca^2+^ signals [3, 7, 8].

Ca^2+^-binding proteins, which contain the conserved EF-hand motif of a characteristic helix-loop-helix motif, have been identified extensively in plant genomes [7–9]. Calmodulin-like proteins (CMLs) are a large subgroup of plant-specific Ca^2+^ sensors and are the key components in Ca^2+^ signal transduction [7, 8]. CMLs are restricted to plants and differ from Calmodulin (CaM), which is the highly evolutionarily conserved Ca^2+^-binding protein in eukaryotes [10, 11]. CaM is composed of four EF-hand domains in plants, whereas CMLs normally possess one to six EF-hands and share 16–75% amino acid identity with CaM [9, 11]. Sequence divergences of EF-hand motifs in CML proteins contribute to the reception of Ca^2+^ signals from different stimuli and to recognition and activation of the target [3, 8]. Many genes encoding CMLs have been characterized and analysed in the genomes of many plant species. To date, a total of 50 and 32 *CML* genes have been identified in the model plant *Arabidopsis* and rice, respectively [12–14]. Additionally, lists of *CML* genes were discovered in some vegetable crops, such as tomato, cucumber and common bean [15, 16].

Previous studies suggest that different *CML* genes likely have distinct and significant physiological roles in a series of developmental processes [8, 9]. *CML24* gene has functions in ion-homeostasis, photoperiod-response and hormone-induced morphogenesis and growth [17]. *Arabidopsis CML23* and *CML24* play roles in the transition to flowering of *Arabidopsis* [17, 18]. Notably, many *CML* genes are crucial for the growth of pollen and pollen tubes [19–22]. For example, the loss-of-function of *CML24* and *CML25* in *Arabidopsis* mutants strongly affected pollen germination and pollen tube growth [22, 23]. Moreover, *CMLs* acting as key players in Ca^2+^ signalling are also involved in the complex signalling pathways responding to abiotic and biotic stresses [8]. In *Arabidopsis*, *AtCML39* is implicated in the transduction of light signals and the promoting of seedling establishment [24]. The knockout mutants of *AtCML9* displayed functions that increased plant tolerance to drought and salinity stress [25]. The *CML24* gene also functions in the inhibition of pathogen-induced NO generation [26]. Furthermore, many reports have suggested the roles of *CMLs* in hormone homeostasis and signalling [3, 8].

Chinese cabbage (*Brassica rapa* L. ssp*. pekinensis*), a member of the Brassicaceae family, is an important leaf vegetable crop that is grown worldwide. A comparative genomic analysis revealed the close relationship between Chinese cabbage and *Arabidopsis*. The whole genome data of Chinese cabbage provide useful resource for the analysis of Ca^2+^-binding proteins including, CaM, CML and calcium-dependent protein kinase (CDPK) in Chinese cabbage [16, 27]. Although 36 *CML* genes have been characterized in *B. rapa* using the BLASTP program [16], some potential candidate *CML* genes and their roles require further exploration. To date, systematic expression analyses of *CMLs* have not been conducted in Chinese cabbage. Therefore, the comprehensive identification and expression analysis of *CML* genes in Chinese cabbage could uncover the molecular mechanisms responsible for responses to abiotic and biotic stresses. In this study, to further systematically explore the *CML* genes in Chinese cabbage, genomic analysis and similarity searching of the conserved EF-hand domain against the whole genome of Chinese cabbage were performed. The primary aims of this study were to identify the potential *BrCML* genes at the whole-genome level and then analyse their gene structure, chromosomal distribution and orthologous genes. Moreover, the expression profiles of *BrCML* genes were investigated in different tissues and under various stress treatments. A putative interaction network of BrCML proteins was proposed to explore their roles in plant development and stress responses in Chinese cabbage. The outcomes of this study provide insights into the understanding of *BrCML* genes in Chinese cabbage and should facilitate the discovery of more *CML* genes in other Brassicaceae crops.

## Results

### Identification and characterization of *BrCML* genes in Chinese cabbage

To identify the putative *CML* genes of Chinese cabbage, 50 CML protein sequences of *Arabidopsis* [12] were retrieved and used as the query to search against BRAD and NCBI databases using the BLASTP program. As a result, 79 protein sequences in Chinese cabbage were obtained and subjected to Pfam, SMART analysis and InterProScan sequence searching. These identified genes were named, in order, from *BrCML1* to *BrCML50* (Additional file 1: Table S1). Among these *BrCML* genes, *BrCML8–2*/*BrCML8–3*, *BrCML11–2*/*BrCML11–3*, *BrCML22–1*/*BrCML22–2* and *BrCML41–1*/*BrCML41–2* were different transcript variants or isoforms from the same gene. The length of protein sequences ranged from 100 (BrCML9–2) to 411 (BrCML50–2) amino acids. Furthermore, the physical and chemical characteristics of 79 *BrCML*s were analysed (Additional file 1: Table S1). The molecular weights of the proteins ranged from 11.078 to 43.862 kDa, and the theoretical pI values ranged from 4.02 to 9.02. More detailed information, including the instability index, aliphatic index and grand average of hydropathicity (GRAVY), is provided. Additionally, compared with the previous report, we found that 22 *BrCML* genes were same as the results by Mohanta et al. [16], and the repeated gene were marked and listed in Additional file 1: Table S1.

**Fig. 1:**
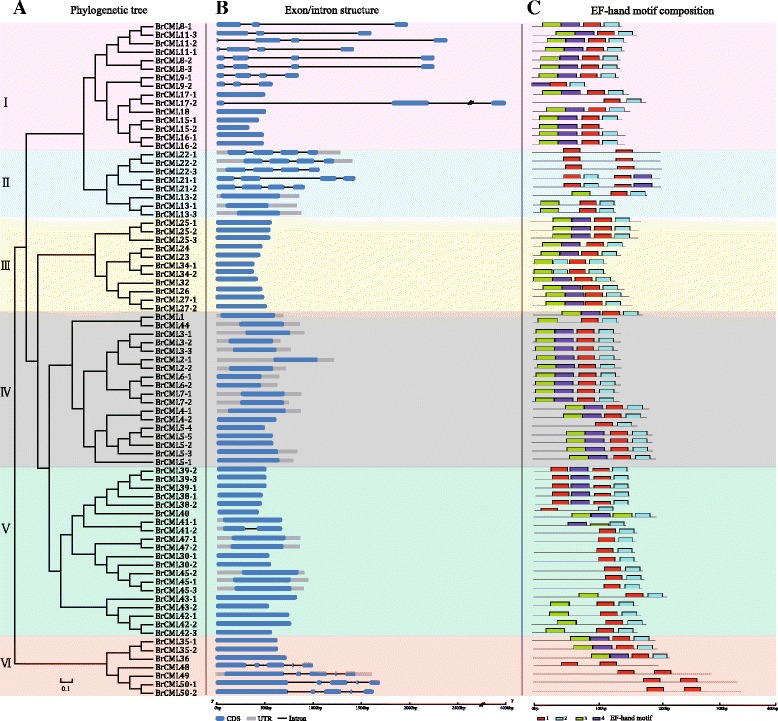
Characterization of the identified *BrCMLs* in Chinese cabbage. **a** Phylogenetic relationships and classification of BrCML proteins. **b** The exon-intron structure distribution of *BrCML* genes. **c** Distribution of conserved EF-hand motifs among the BrCML proteins

To obtain the characterization and subfamily classification of the 79 BrCML proteins, their amino acid sequences were used to generate a phylogenetic tree by the NJ Bootstrap method (Fig. 1a). All BrCMLs were classified into six subfamilies (groups I-VI). The sequence similarities of members in each subgroup were relatively high. Moreover, the exon-intron structures were analysed to examine the structural diversity of *BrCML* genes (Fig. 1b). Most of the genes had a single exon without intron region, particularly some of the genes in groups III, IV and V. To further analyse the features of the 79 BrCML proteins, the MEME tool was used to search and predict their conserved domains. As the results display, the BrCML proteins contained two to four highly conserved EF-hand motifs (Fig. 1c). The LOGO of four amino acid motifs was also generated (Additional file 2: Figure S1). In general, the genes in the same subgroup shared a close phylogenetic relationship, high sequence similarity and similar gene structures.

### Phylogenetic relationships of CMLs among Chinese cabbage, *Arabidopsis* and rice

**Fig. 2:**
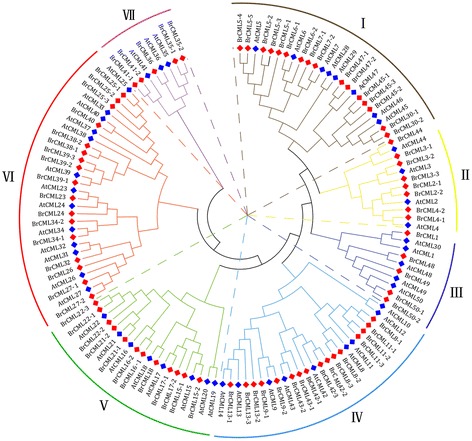
Phylogenetic tree of CML proteins in Chinese cabbage and *Arabidopsis* constructed with the neighbourhood-joining method

The protein sequences of the 79 BrCMLs in Chinese cabbage and 50 AtCMLs in *Arabidopsis* were obtained in this study. The evolutionary relationships and classification of CMLs between Chinese cabbage and *Arabidopsis* were analysed based on the full-length amino acid sequences (Fig. 2). The constructed phylogenetic tree showed that the CML proteins were classified into seven subgroups (groups I-VII). Most CMLs were in group VI, followed by groups IV andI, with the relatively few genes in groups II, III and VII. Notably, group VII was the smallest group and contained only five BrCMLs and three AtCMLs. Moreover, the amino acid sequences of 32 OsCMLs on rice were downloaded from the rice genome database (TIGR), and a more detailed phylogenetic analysis of CMLs was performed among Chinese cabbage, *Arabidopsis* and rice (Additional file 3: Figure S2). The evolutionary relationships suggested that most *BrCML* genes were closely related to their corresponding homologous genes in *Arabidopsis* and rice.

### Chromosomal localization and orthologous gene analysis of *BrCML* genes

**Fig. 3:**
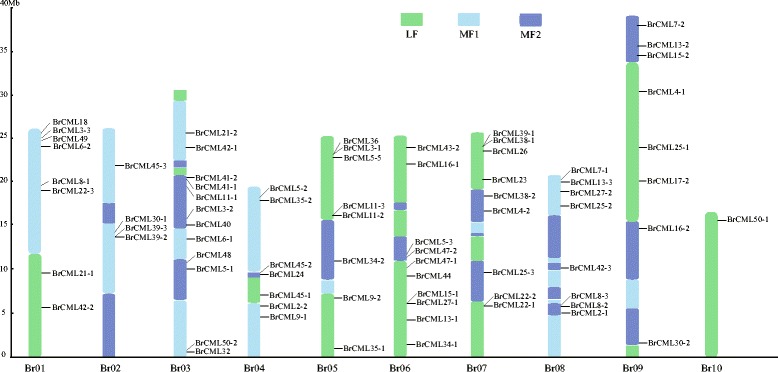
Chromosome distribution of *BrCML* genes in Chinese cabbage. LF: the least fractionated subgenome; MF1: the medium fractionated subgenome; MF2: the most fractionated subgenome

**Fig. 4:**
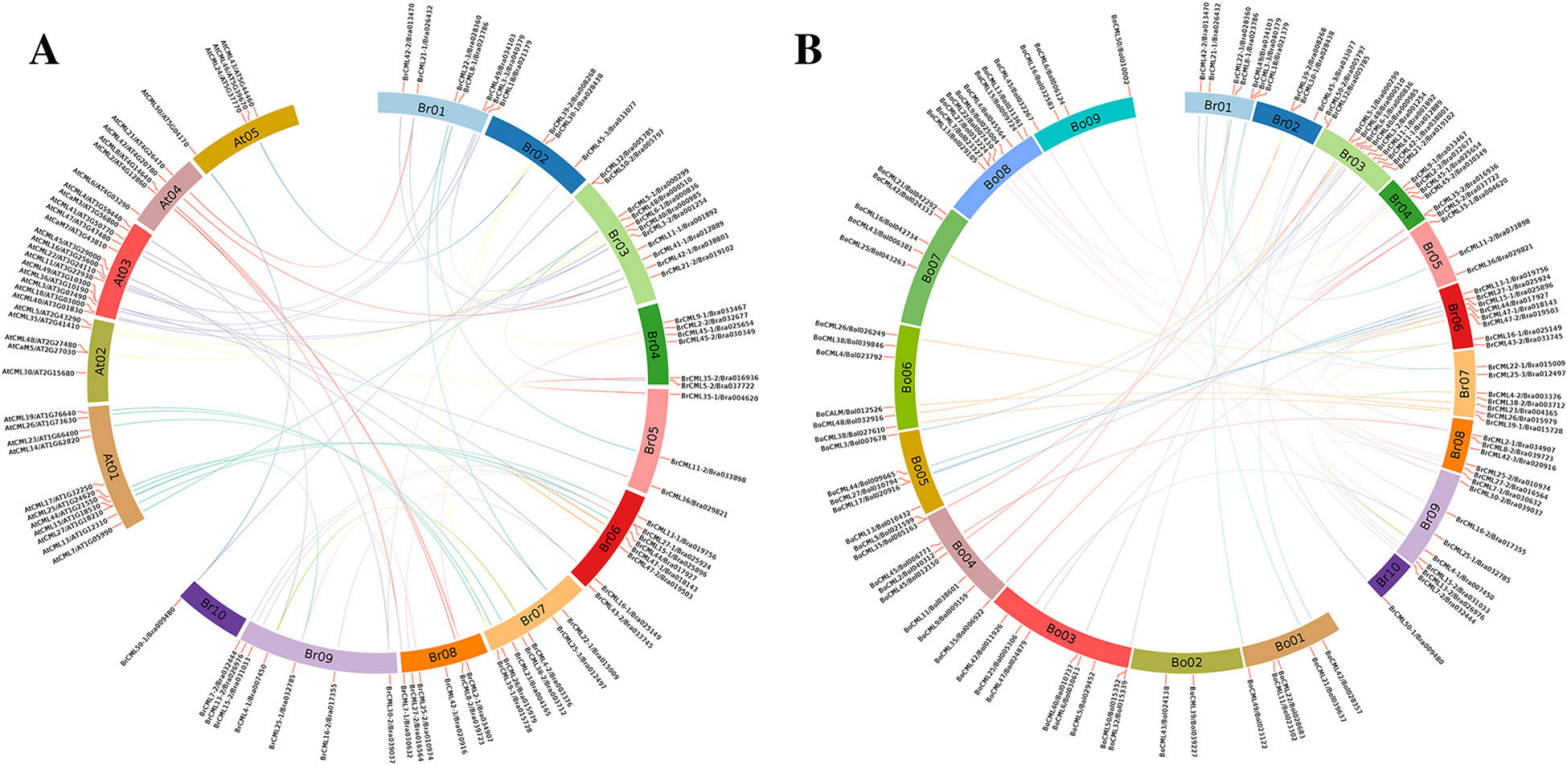
Syntenic analysis of *CML* genes in Chinese cabbage, *Arabidopsis thaliana* and *Brassica oleracea.* **a** The orthologous and paralogous *BrCML* genes were mapped onto the chromosomes of Chinese cabbage (Br01-Br10) and *Arabidopsis* (At01-At05). **b** The orthologous and paralogous *BrCML* genes were mapped onto the chromosomes of Chinese cabbage (Br01-Br10) and *Brassica oleracea* (Bo01-Bo09)

To examine the chromosomal distribution of the 79 *BrCMLs*, the genes were mapped onto the chromosomes of Chinese cabbage against the *B. rapa* genome database (chromosome v1.5). In total, 75 *BrCML* genes were separately distributed on the ten chromosomes (Fig. 3), whereas four genes (*BrCML3–1*, *BrCML6–2*, *BrCML13–3* and *BrCML17–2*) could not be assigned to any specific chromosome. Most *BrCML* genes were located on chromosome Br03, whereas a single *BrCML50–1* was found on chromosome Br10. Furthermore, the *BrCML* genes were anchored on the three fractionated subgenomes of the *B. rapa* genome, including the least fractionated (LF) subgenome, the medium fractionated (MF1) subgenome and the most fractionated (MF2) subgenome [28], with 27 *BrCMLs* fractionated into the LF subgenome, 23 into the MF1 subgenome, and 25 into the MF2 subgenome (Fig. 3; Additional file 4: Table S2). Moreover, 21 pairs of *BrCML* syntenic paralogs were found on different subgenomes of Chinese cabbage (Additional file 4: Table S2). For example, *BrCML15–1*/Bra025896 and *BrCML15–2*/Bra031033 were located in the LF and MF2, respectively, and both exhibited high sequence similarities with *AtCML15* (AT1G18530). The syntenic analysis of these *BrCML* paralogs was performed, and the results were shown in Fig. 4.

Furthermore, in this study, comparative analysis was used to identify orthologous *CML* genes among Chinese cabbage, *A. thaliana* and *B. oleracea*. As a result, 64 and 62 pairs of orthologous *CMLs* were identified between Chinese cabbage and *A. thaliana* and between Chinese cabbage and *B. oleracea*, respectively (Fig. 4; Additional file 5: Table S3). Among these orthologous *CMLs*, most *BrCML* genes were found with 1–6 orthologous genes in *A. thaliana* and *B. oleracea*. Three genes (*BrCML9–1*, *BrCML24* and *BrCML32*) had no orthologue in *Arabidopsis*, and two genes (*BrCML8–2* and *BrCML17–1*) had no orthologue in *B. oleracea*. Additionally, several orthologous genes of *BrCML8–1* and *BrCML8–2* belonged to *Arabidopsis CaM* genes (Additional file 5: Table S3), suggesting the close genetic relationship and most likely similar function between *CML* and *CaM* genes. The orthologous genes should provide a strong resource and reference for exploring the functions and roles of *BrCMLs* in Chinese cabbage.

### Expression profiling of *BrCML* genes in different tissues and under stress treatments

**Fig. 5:**
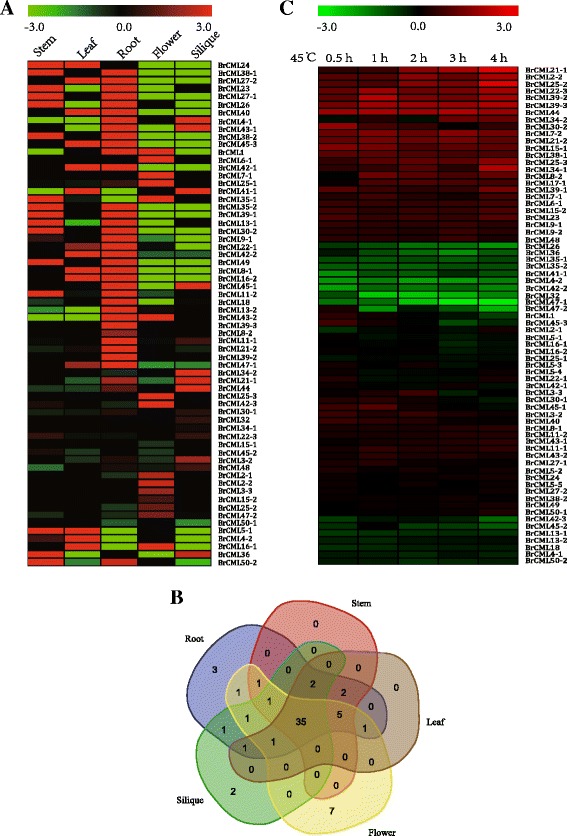
Expression analysis of *BrCML* genes in Chinese cabbage. **a** Differential expression patterns of *BrCML* genes in root, stem, leaf, flower and silique of Chinese cabbage. **b** Venn diagram of *BrCML* expressions in five tissues of Chinese cabbage. **c** Differential expression patterns of *BrCML* genes under 45 °C heat stress for 0.5, 1, 2, 3 and 4 h

To detect the tissue-specific expression profiling of the *BrCML* genes, the gene expression FPKM values of 71 sequences in five different tissues were calculated by exploiting the previously reported RNA-Seq data on Chinese cabbage ‘Chiifu’ [29]. The map of the expression profiling for the *BrCMLs* was prepared using the log_2_FPKM values (Fig. 5a). The *BrCML* genes exhibited differential expression in root, stem, leaf, flower and silique of Chinese cabbage, although the expression of eight genes was not found in any tissue. *BrCML24* had the highest levels of expression in stem, leaf, flower and silique and was second only to *BrCML38–1* in the root (Additional file 6: Table S4). Venn diagram of the expression analysis revealed that 35 of the *BrCMLs* were expressed in five tissues, whereas several genes were specifically expressed in a single tissue (Fig. 5b). For example, *BrCML8–2*, *BrCML39–2* and *BrCML39–3* were only expressed in the root, and *BrCML32* and *BrCML34–1* were only expressed in the silique. Remarkably, seven genes (*BrCML2–2*, *BrCML3–3*, *BrCML6–1*, *BrCML7–1*, *BrCML15–1*, *BrCML15–2* and *BrCML25–3*) were only expressed in flower and considered as flower-specific expressions, implying that they play vital roles in the development of floral organs or other relevant biological processes.

The relative expression levels of all the identified *BrCML* genes were also validated under temperature stress. In this study, the expression values of *BrCML* genes under 45 °C heat stress were obtained from a microarray study with the Chinese cabbage ‘Chiifu’ [30]. A total of 71 *BrCML* genes were analysed and their fold changes under different treatments were counted (Additional file 7: Table S5). These genes showed diverse dynamic expression patterns in response to 45 °C heat stress with exposure at 0.5, 1, 2, 3 or 4 h (Fig. 5c). In the five treatment groups under 45 °C heat stress, the expression of 38 and 18 *BrCML* genes was up-regulated and down-regulated, respectively. Differential expression levels of most genes were less than 2-fold under treatment (Additional file 7: Table S5). Furthermore, the differential expression of *BrCML* genes related to the formation of leafy-head in response to low temperature was analysed. The transcriptomic data of Chinese cabbage inbred line ‘Chiifu-402’ were retrieved from a previous report concerning low-temperature induced leafy-head formation [31]. Consequently, 54 differentially expressed *BrCML* genes were detected between the heading group (a constant 25 °C temperature) and non-heading group (a 4 °C low temperature treatment) (Additional file 8: Figure S3; Additional file 9: Table S6).

### Identification of miRNAs targeting *BrCML* genes

MiRNAs are known to regulate the expression of corresponding target genes [32]. To identify the putative miRNAs targeting *BrCML* genes, the data of several miRNA libraries were obtained from previous reports on miRNA identification in Chinese cabbage. The psRNATarget program performed the target prediction of miRNAs. In this study, six known miRNAs and one novel miRNAs (named miRN01) were predicted to potentially target 10 *BrCML* genes (Additional file 10: Table S7). The putative regulatory relationships of 10 miRNA-*BrCML* pairs (Additional file 11: Figure S4) were described using Cytoscape software. Moreover, miR835 and miR838 separately targeted two different *BrCML* genes, and miRN01 targeted two syntenic genes of *BrCML25*.

### The interaction networks of BrCML proteins in Chinese cabbage

**Fig. 6:**
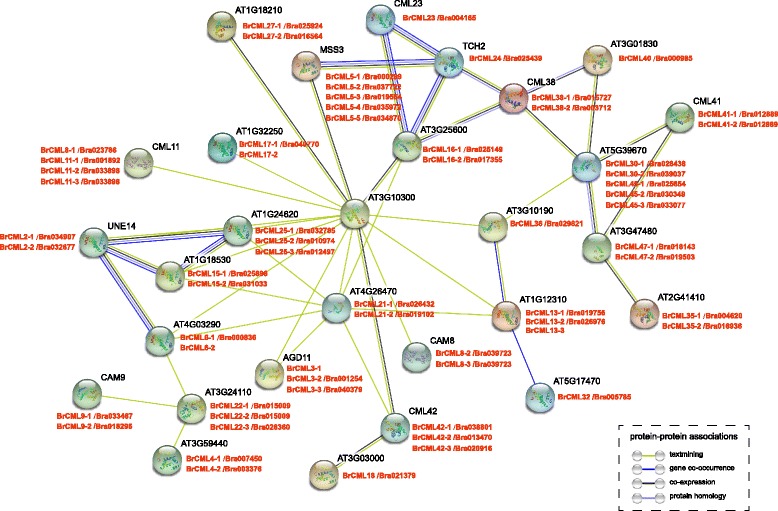
The putative interaction network of CML proteins. The homologous genes from Chinese cabbage and *Arabidopsis* are in red and black, respectively

In this study, 79 BrCML proteins were screened in Chinese cabbage, most of which shared close relationships with their homologs or orthologues in *Arabidopsis*. To validate the protein interactions of BrCMLs, STRING software was used to construct a putative interaction network according to the orthologous proteins in *Arabidopsis* (Fig. 6). A total of 63 BrCML proteins associated with 28 known *Arabidopsis* proteins were involved in the network, whereas 15 BrCMLs and 9 proteins of *Arabidopsis* were not found in associations with any other proteins (Fig. 6; Additional file 12: Table S8). A central node (AT3G10300) was associated with the most proteins, including 38 BrCML and 15 *Arabidopsis* CML proteins. The protein-protein associations revealed that some BrCML proteins were most likely co-expressed according to *Arabidopsis* CML proteins. Of note, BrCML2, BrCML6, BrCML15 and BrCML25 formed a close protein interaction and showed putative co-expression and co-occurrence (Fig. 6). The analysis of interaction network suggested that several BrCML proteins could interact with one another and regulate downstream proteins, thereby, enhancing the understanding of BrCML protein functions in Chinese cabbage.

## Discussion

Plant Ca^2+^ sensor proteins involved in Ca^2+^ signalling networks are crucial proteins governing Ca^2+^ homeostasis during multiple cellular processes [1, 2]. The CMLs are a primary group of unique EF-hand proteins in plants that bind to Ca^2+^ and regulate downstream targets in response to various stimuli-induced Ca^2+^ fluctuations and signalling transduction [3, 6, 8]. A series of CML proteins and an increasing number of target genes have been identified in many plant species. For example, 50 and 32 CML genes have been found in *Arabidopsis* and rice, respectively [12–14]. Chinese cabbage is one of the important leaf vegetables with high nutritional and economic value and is widely grown around the world. Nevertheless, the characterization and expression analysis of *BrCML* genes in Chinese cabbage have not been studied. In this study, homology searching against the BRAD and the NCBI database was performed and 79 *BrCML* genes were identified in Chinese cabbage, a number comparable with that in previous reports in the model crop *Arabidopsis* [12, 13, 16]. To obtain a comprehensive overview and characterization of these *BrCML* genes, the biochemical properties, structural features and expression patterns were explored to provide a rich source for further functional studies of *BrCMLs* in Chinese cabbage.

As demonstrated by the evolutionary conservation in multiple species, CaM proteins have important functions in response to different developmental and environmental signals [10, 11, 13]. Plant CMLs have at least 16% sequence identity with the conserved CaM proteins and display a diversity of protein structures and expression features [8, 9, 12]. The structural variation of CML proteins may confer functional diversity, and the number and sequence variation of EF-hands among CML members are implicated in the Ca^2+^ binding properties and differential responses [3, 10, 13]. In this study, two to four conserved EF-hand motifs were typically found in the identified *BrCML* genes (Fig. 1c), which is consistent with the typical character of CML proteins [10]. The intron-exon structure analysis showed that most *CML* genes were intronless, which is in agreement with the previous reports [9, 12, 16]. Some *BrCML* genes that shared close phylogenetic relationships displayed similar EF-hand distributions and intron-exon structures, which indicate that these genes may have similar biological roles and expression characteristics. Phylogenetic analysis of *CML* genes between Chinese cabbage and *Arabidopsis* showed that many *BrCML* genes had high similarities with their corresponding *Arabidopsis* homologs. Moreover, most of the identified *BrCML* genes were found as orthologous genes in *Arabidopsis* and *B. oleracea*, suggesting the conservation of *CML* genes among Brassicaceae crops. The current evidence indicates that *Brassica* species underwent an ancestral whole-genome triplication from their diploid progenitors [33, 34]. Comparative analysis of *Brassica* genomes demonstrates that the *B. rapa* genome evolved from a hexaploid progenitor with a triplicated diploid ancestral genome [28, 34]. In the present study, we assigned the 79 *BrCML* genes across the three different subgenomes of Chinese cabbage (Fig. 3), which showed a relatively uniform distribution with 27 genes in the LF, 23 in the MF1 and 25 in the MF2. We also identified 21 pairs of *BrCML* syntenic paralogs, supporting the extensive genome triplication of Chinese cabbage [28].

The spatio-temporal expression pattern of CMLs is considered a critical index for their function annotations [3, 8]. Many reports demonstrate that *CML* genes are differentially expressed in different plant tissues, developmental stages and cell type and in response to various external stimuli [13, 35]. Expression profiling of *CML* genes is extensively validated in many species, including pea, tomato, strawberry, *Arabidopsis* and rice [15, 19, 35–37]. As expected, our results revealed that the expression of *BrCML* genes differed distinctly among five Chinese cabbage tissues, with several *BrCML* genes exhibiting tissue-specific expression patterns (Fig. 5). Notably, the expression of *BrCML2–2*, *BrCML6–1*, *BrCML15–1*, *BrCML15–2* and *BrCML25–3* was specific to the flower of Chinese cabbage. Moreover, the interaction network of *BrCML* genes suggested that BrCML2, BrCML6, BrCML15 and BrCML25 were co-expressed based on reference to the homologs in *Arabidopsis*, implying that these BrCMLs may participate in the same regulatory pathways and are capable of similar functions during plant development and stress responses. Transcriptomic analysis of *Arabidopsis* showed that a set of *CMLs* (e.g., *CML2*, *CML6*, *CML15*, *CML25*, *CML42* and *CML49*) were significantly up-regulated during pollen germination or pollen tube growth [17]. Furthermore, *AtCML25* mediates the regulation of Ca^2+^ and K^+^ transmembrane trafficking in pollen grains and pollen tubes and acts as an important transducer during pollen germination and tube elongation [21]. This evidence could support a hypothesis that BrCML2, BrCML6, BrCML15 and BrCML25 may exert functions in pollen germination and pollen tube growth of Chinese cabbage. Additionally, in this study, the candidate miRN01 possibly negatively targeted *BrCML25–1*/Bra032785 and *BrCML25–2*/Bra010974 (Additional file 10: Table S7). Functional analysis of miRNAs could greatly increase the understanding of *CML* gene functions.

The expression of *CaM* and *CML* genes, and alteration of intracellular Ca^2+^ gradients, varies specifically in response to Ca^2+^ signalling and a variety of stress responses, including biotic and abiotic stresses [7, 8, 13]. In *Arabidopsis*, *AtCML37*, *AtCML38* and *AtCML39* respond to several developmental and stimulus-induced signalling pathways [23, 38], and *AtCML42* and *AtCML43* may function to increase resistance to pathogens [39, 40]. Additionally, *CaM1* in pepper and *CaM13* in tobacco play vital roles in virus-induced plant immune responses [41, 42]. The mutant of *AtCaM3* showed reduced thermotolerance and the down-regulation of heat-related proteins [43, 44]. According to the summary of Delk et al. [17], the expression levels of *CML24* are related to responses to hormones, cold, and heat, among others. In this study, 45 °C heat stress induced an increase in transcript levels of many *BrCML* genes, particularly for *BrCML21–1*, which exhibited the highest fold change in expressions. Moreover, *BrCML44* showed reverse patterns of expression under 45 °C and 4 °C treatments, indicating that the expression was susceptible to heat stress or low temperature. These results will contribute to further functional explorations of *BrCML* genes in Chinese cabbage.

## Conclusions

In this study, a total of 79 *BrCML* genes were identified in Chinese cabbage at the whole-genome level. Comprehensive analysis and expression profiling of *BrCML* genes were performed to determine the potential functions in Ca^2+^ signalling networks and in response to various stresses and environmental stimuli. The *BrCML* genes contained two to four conserved EF-hand motifs and shared close relationships with their homologs in *Arabidopsis*. We also found a series of orthologous *BrCML* genes in *Arabidopsis* and *B. oleracea*. Furthermore, expression analysis revealed the tissue-specific expression and temperature susceptibility of *BrCML* genes. Several genes, including *BrCML2–2*, *BrCML6–1*, *BrCML15–1*, *BrCML15–2* and *BrCML25–3*, showed flower-specific expression patterns. Remarkably, a protein interaction analysis showed that BrCML2, BrCML6, BrCML15 and BrCML25 were likely co-expressed, suggesting that these proteins have critical functions in biological processes relevant to Chinese cabbage flower development. These results will provide a rich resource for further functional studies of BrCML proteins in Chinese cabbage.

## Methods

### Identification of *BrCML* genes in Chinese cabbage

To identify the *CML* genes in Chinese cabbage, the reported CML gene sequences in *Arabidopsis thaliana* were downloaded from the TAIR database (The Arabidopsis Information Resource) and used as a query to perform BLASTP searching. The whole genome sequences of Chinese cabbage from the BRAD database (*Brassica* database) [45] and the NCBI database were used to search the potential *CML* genes in Chinese cabbage. To differentiate between *CaM* and *CML* genes which were homologs in Chinese cabbage, we follow the principle of the major character of EF-hand containing proteins [9, 11, 15, 16, 37] and screen the candidate *CML* genes. The candidate proteins of *CML* genes were further confirmed by analysing the EF-hand motifs through the use of public databases including NCBI Conserved Domain Database (http://www.ncbi.nlm.nih.gov/cdd), Pfam (http://pfam.xfam.org/), InterProScan (http://www.ebi.ac.uk/Tools/pfa/iprscan5/) and SMART (http://smart.embl-heidelberg.de/), and the domain ID of searching EF-hand was cd00051, PF00036, IPR002048 and SM00054 in each database, respectively. The identified candidate genes were named from *BrCML1* to *BrCML50* according to homologous genes and annotation descriptions. Their gene sequences and deduced amino acid sequences were retrieved for the following characterization of *BrCML* genes.

### Analysis of conserved domain, gene structure and characterization of *BrCML* genes

The BrCML protein sequences were analysed for physical and chemical characteristics, including the molecular weight (MW), theoretical point (pI), instability index, aliphatic index and grand average of hydropathicity (GRAVY), using the ProtParam tool of ExPASy (http://web.expasy.org/protparam/). The exon-intron structure analysis of *BrCML* genes was conducted using the GSDS 2.0 (Gene structure display server; http://gsds.cbi.pku.edu.cn//index.php) program with default parameters. The conserved motifs were analysed using the MEME tool (version 4.11.4; http://meme-suite.org/tools/meme), with the minimum width of motifs as 10, the maximum width of motifs as 40 and the other parameters as default values. The LOGO of conserved motifs was described according to the motif analysis results.

### Phylogenetic relationships of CML proteins in Chinese cabbage, *Arabidopsis* and rice

The multiple alignments of BrCML proteins were performed using ClustalX with default parameters. The reported 32 OsCMLs in *Oryza sativa* L. [14] were downloaded from the rice genome database (The Institute for Genomic Research, TIGR; http://rice.plantbiology.msu.edu/). Phylogenetic analysis of CML proteins in Chinese cabbage, *Arabidopsis* and rice was performed using MEGA 7.0 [46] with the neighbourhood-joining (NJ) method and bootstrap values of 1000 replicates.

### Identification of orthologous *BrCML* genes and syntenic analysis in Chinese cabbage

According to the BRAD and chromosome data (v1.5) of Chinese cabbage, the identified *BrCML* genes were located into ten chromosomes and three fractionated subgenomes. Chromosomal positions of *BrCML* genes were analysed and presented using MapInspect Software. The syntenic genes of *BrCMLs* were identified in online searching on the BRAD (http://brassicadb.org/brad/searchSynteny.php). The orthologous *BrCML* genes among Chinese cabbage, *A. thaliana* and *Brassica oleracea* were also searched. Cricos software [47] was employed for syntenic analysis of orthologous and paralogous *CML* genes among Chinese cabbage, *A. thaliana* and *B. oleracea.*

### Expression pattern analysis of *BrCML* genes and interaction networks

To analyse the expression patterns of *BrCML* genes in Chinese cabbage, the Illumina RNA-Seq data of *B. rapa* ‘Chiifu’ [29] were downloaded and used for gene expression profiling in five tissues that included root, stem, leaf, flower and silique. The Fragments Per kb per Million reads (FPKM) values [48] were used to present the *BrCML* expression levels. The expression values of genes with abundance of zero were modified to 0.001 for further analysis. The genes with fold change greater than or less than zero were determined as up- or down-expressed genes, respectively. Fold change was calculated as log_2_ (FPKM in treatment / FPKM in control). Moreover, the microarray and transcriptomic data of Chinese cabbage ‘Chiifu’ under 45 °C heat stress [30] and low temperature treatment [31] were obtained from previous reports, respectively. More than two replications were performed in expression analysis. The gene expression levels from different replicates in these papers were calculated according to the previously reported method by Yanai et al. [49]. Clustering analysis of *BrCML* genes was performed using Cluster software [50]. The heat maps of gene expressions were visualized by Java Treeview software [51]. STRING software (http://string-db.org/) [52] was used to generate the interaction networks of *Arabidopsis CML* genes. The putative interaction networks of *BrCML* genes were constructed according to the corresponding homologs between Chinese cabbage and *Arabidopsis*.

### Prediction of microRNAs targeting *BrCML* genes

The data of several miRNA libraries were obtained from previous reports on Chinese cabbage [53–56]. The psRNATarget (http://plantgrn.noble.org/psRNATarget/) [57] program was used to predict the putative miRNAs targeting *BrCML* genes. The putative regulatory relationships between miRNAs and *BrCMLs* were analysed and visualized using Cytoscape software [58].

## Additional files

Additional file 1: Table S1. The information on *BrCML* genes in Chinese cabbage. (XLSX 22 kb)

Additional file 2: Figure S1. The LOGO of four conserved EF-hand motifs among BrCML proteins. (PDF 412 kb)

Additional file 3: Figure S2. Phylogenetic analysis of CML proteins in Chinese cabbage, *Arabidopsis* and rice. (PDF 742 kb)

Additional file 4: Table S2. The distribution and syntenic paralogs of *BrCML* genes in three subgenomes of Chinese cabbage. (XLSX 9 kb)

Additional file 5: Table S3. The orthologous gene pairs (*E*-value ≤1E − 10) in CML proteins of Chinese cabbage, *Arabidopsis thaliana* and *Brassica oleracea. (XLSX 12 kb)*

Additional file 6: Table S4. The FPKM values of *BrCML* genes in five different tissues of Chinese cabbage. (XLSX 13 kb)

Additional file 7: Table S5. The expression values of *BrCML* genes under 45 °C heat treatment in Chinese cabbage. (XLSX 19 kb)

Additional file 8: Figure S3. Differential expression patterns of *BrCML* genes under low temperature stress. ‘Heading’: the group with a constant 25 °C temperature; ‘non-heading’: the group with a 4 °C low temperature treatment. (PDF 407 kb)

Additional file 9: Table S6. The expression values of *BrCML* genes under low temperature treatment in Chinese cabbage. (XLSX 11 kb)

Additional file 10: Table S7. The putative miRNAs targeting *BrCML* genes from previously reported libraries. (XLSX 9 kb)

Additional file 11: Figure S4. Putative regulatory relationships between candidate miRNAs and *BrCML* genes. (PDF 76 kb)

Additional file 12: Table S8. Information of STRING search results for interaction networks of *BrCML* genes. (XLSX 12 kb)

## Abbreviations

BRAD: Brassica database
CaM: Calmodulin
CML: Calmodulin-like protein
FPKM: Fragments Per kb per Million reads
LF: The least fractionated subgenome
MF1: The medium fractionated subgenome
MF2: The most fractionated subgenome
NJ: Neighbourhood-joining method
