# Supplementary figures and images for: Genome-wide identification and expression analysis of calmodulin-like (CML) genes in Chinese cabbage (Brassica rapa L. ssp. pekinensis)
Source: BMC Genomics. 2017 Nov 2;18:842. doi: 10.1186/s12864-017-4240-2 (PMC5668983; doi:10.1186/s12864-017-4240-2)

|         | Logo                                                                               | Width | Motif Similarity Matrix |      |      |      |
|---------|------------------------------------------------------------------------------------|-------|-------------------------|------|------|------|
|         |                                                                                    |       | 1.                      | 2.   | 3.   | 4.   |
| Motif 1 | 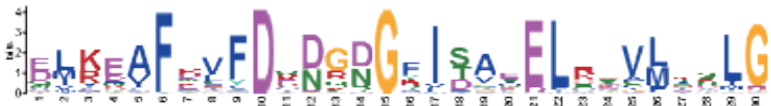 | 30    | --                      | 0.56 | 0.69 | 0.49 |
| Motif 2 | 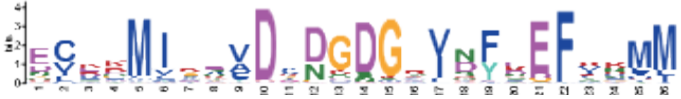 | 26    | 0.56                    | --   | 0.52 | 0.61 |
| Motif 3 | 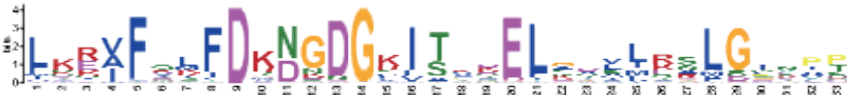 | 33    | 0.69                    | 0.52 | --   | 0.44 |
| Motif 4 | 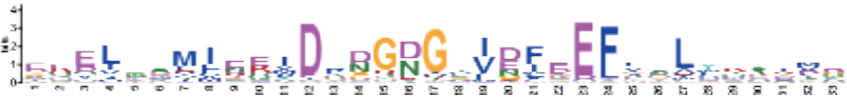 | 33    | 0.49                    | 0.61 | 0.44 | --   |

Supplement: Supplementary file 2 — The LOGO of four conserved EF-hand motifs among BrCML proteins. (PDF 412 kb) [file 12864_2017_4240_MOESM2_ESM.pdf]

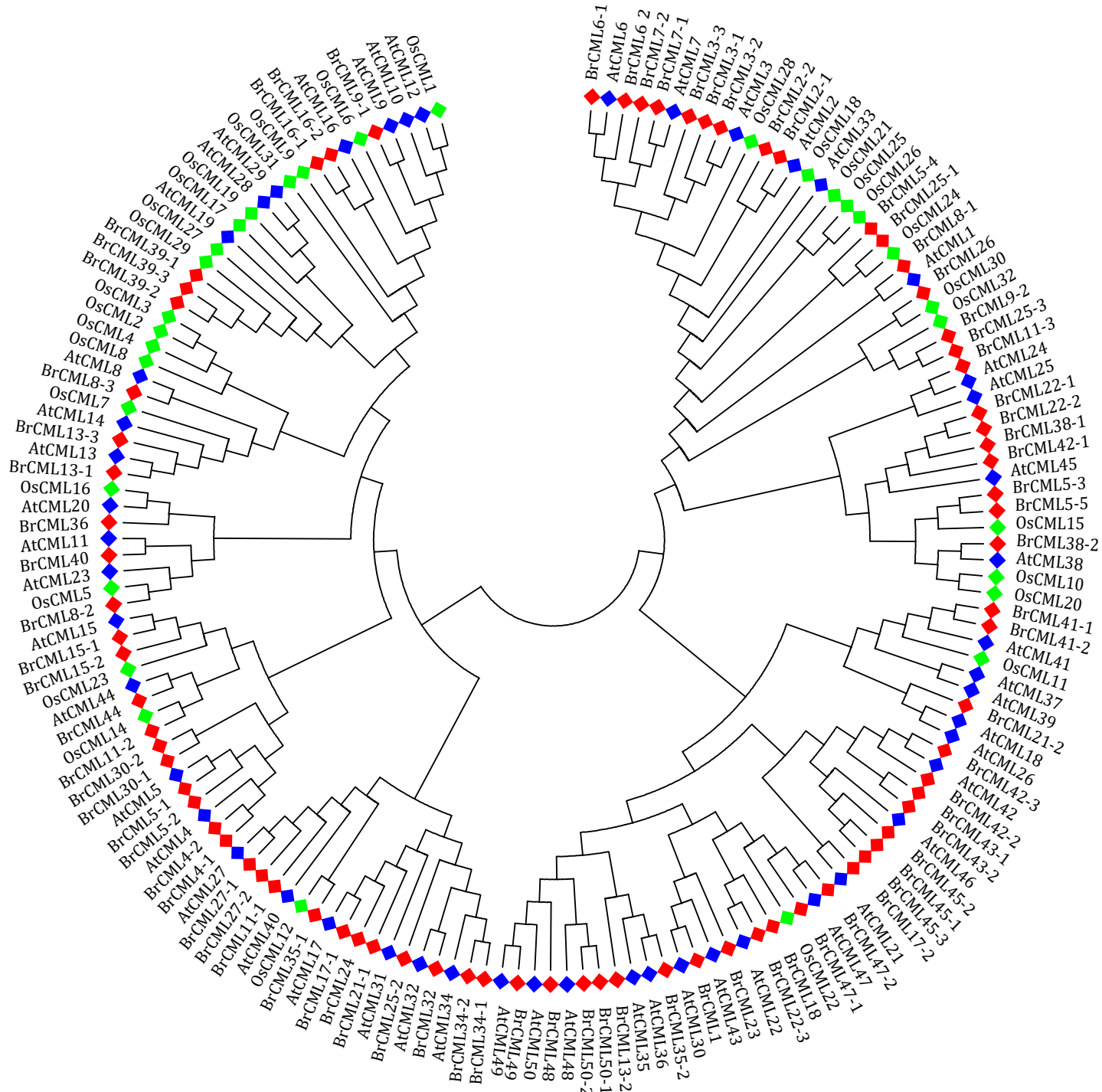

Supplement: Supplementary file 3 — Phylogenetic analysis of CML proteins in Chinese cabbage, Arabidopsis and rice. (PDF 742 kb) [file 12864_2017_4240_MOESM3_ESM.pdf]

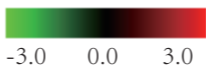

head      non-head

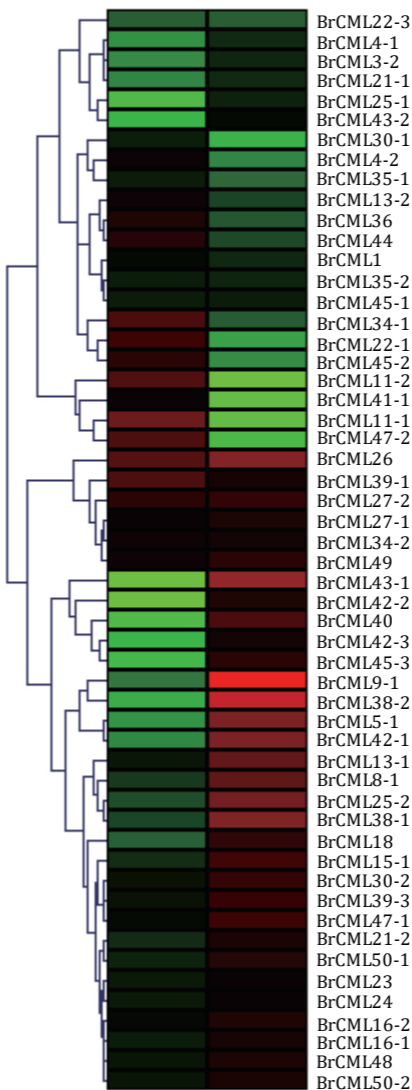

Supplement: Supplementary file 8 — Differential expression patterns of BrCML genes under low temperature stress. ‘Heading’: the group with a constant 25 °C temperature; ‘non-heading’: the group with a 4 °C low temperature treatment. (PDF 407 kb) [file 12864_2017_4240_MOESM8_ESM.pdf]

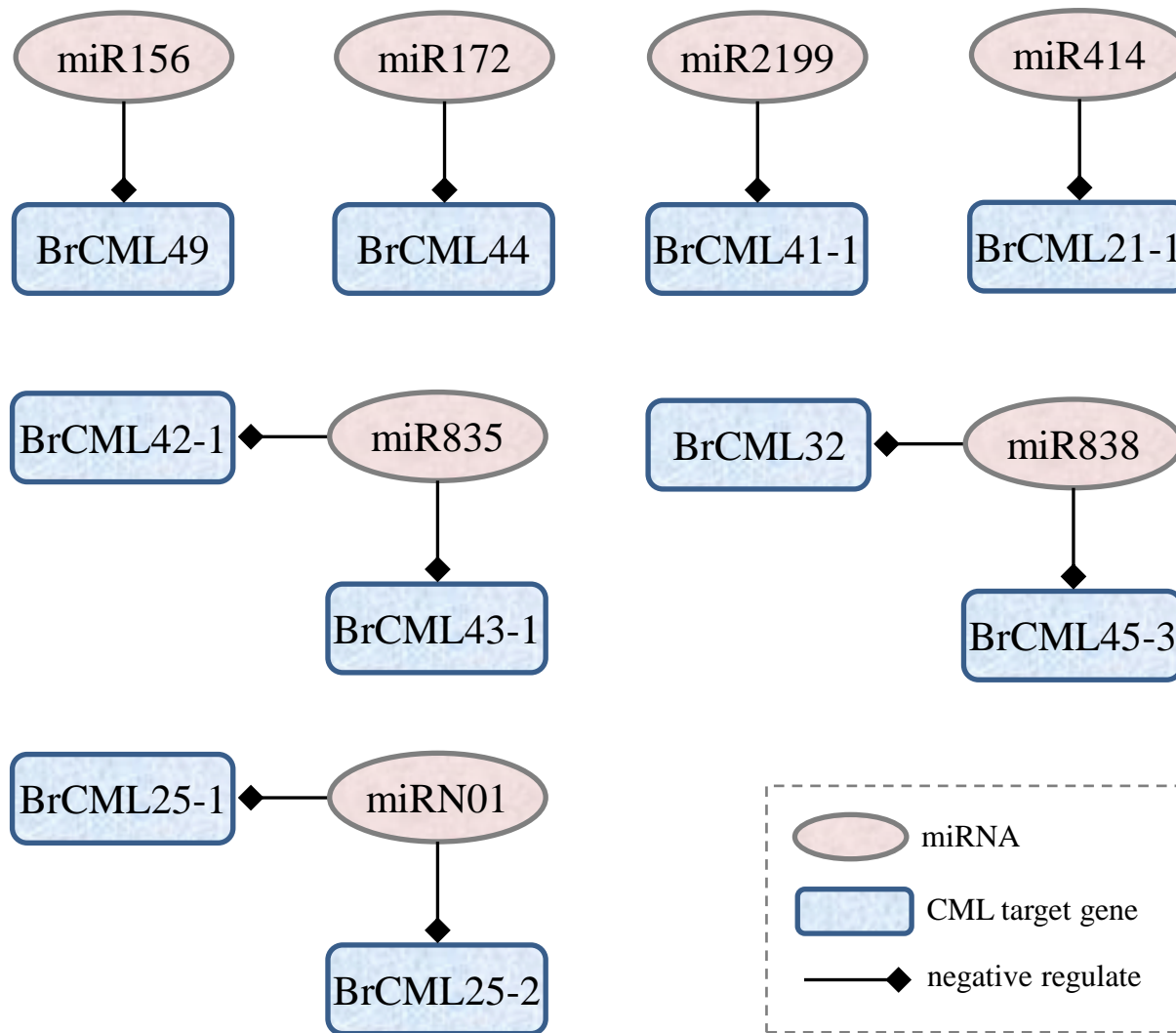

Supplement: Supplementary file 11 — Putative regulatory relationships between candidate miRNAs and BrCML genes. (PDF 76 kb) [file 12864_2017_4240_MOESM11_ESM.pdf]
